# Complete genome sequence of *Riemerella anatipestifer* type strain (ATCC 11845^T^)

**DOI:** 10.4056/sigs.1553862

**Published:** 2011-04-29

**Authors:** Konstantinos Mavromatis, Megan Lu, Monica Misra, Alla Lapidus, Matt Nolan, Susan Lucas, Nancy Hammon, Shweta Deshpande, Jan-Fang Cheng, Roxane Tapia, Cliff Han, Lynne Goodwin, Sam Pitluck, Konstantinos Liolios, Ioanna Pagani, Natalia Ivanova, Natalia Mikhailova, Amrita Pati, Amy Chen, Krishna Palaniappan, Miriam Land, Loren Hauser, Cynthia D. Jeffries, John C. Detter, Evelyne-Marie Brambilla, Manfred Rohde, Markus Göker, Sabine Gronow, Tanja Woyke, James Bristow, Jonathan A. Eisen, Victor Markowitz, Philip Hugenholtz, Hans-Peter Klenk, Nikos C. Kyrpides

**Affiliations:** 1 DOE Joint Genome Institute, Walnut Creek, California, USA; 2 Los Alamos National Laboratory, Bioscience Division, Los Alamos, New Mexico, USA; 3 Biological Data Management and Technology Center, Lawrence Berkeley National Laboratory, Berkeley, California, USA; 4 Oak Ridge National Laboratory, Oak Ridge, Tennessee, USA; 5 DSMZ - German Collection of Microorganisms and Cell Cultures GmbH, Braunschweig, Germany; 6 HZI – Helmholtz Centre for Infection Research, Braunschweig, Germany; 7 University of California Davis Genome Center, Davis, California, USA; 8 Australian Centre for Ecogenomics, School of Chemistry and Molecular Biosciences, The University of Queensland, Brisbane, Australia

**Keywords:** capnophilic, non-motile, Gram-negative, poultry pathogen, mesophilic, chemoorganotrophic, *Flavobacteriaceae*, GEBA

## Abstract

*Riemerella anatipestifer* (Hendrickson and Hilbert 1932) Segers *et al.* 1993 is the type species of the genus *Riemerella*, which belongs to the family *Flavobacteriaceae*. The species is of interest because of the position of the genus in the phylogenetic tree and because of its role as a pathogen of commercially important avian species worldwide. This is the first completed genome sequence of a member of the genus *Riemerella*. The 2,155,121 bp long genome with its 2,001 protein-coding and 51 RNA genes consists of one circular chromosome and is a part of the *** G****enomic* *** E****ncyclopedia of* *** B****acteria and* *** A****rchaea * project.

## Introduction

No strain designation has been published for the type strain of *Riemerella anatipestifer*; therefore it will be referred to in this publication as ATCC 11845^T^, after the earliest known deposit (= DSM 15868 = ATCC 11845 = JCM 9532). Strain ATCC 11845^T^ is the type strain of *R. anatipestifer* which is the type species of the genus *Riemerella*. The organism was described for the first time by Hendrickson and Hilbert in 1932 as '*Pfeifferella anatipestifer*' [1], was subsequently known as '*Pasteurella anapestifer*' [2] and renamed by Bruner and Fabricant in 1954 as *Moraxella anatipestifer* [3,4]. However, since the organism is closely related to neither the genus *Moraxella* nor to *Pasteurella* [5] it was reclassified as a novel genus by Segers *et al*. in 1993 [6]. The reclassification was confirmed by subsequent 16S rRNA gene sequence analysis [7,8]. The generic name was given in honor to Riemer, who first described *R. anatipestifer* infections in geese in 1904 and referred to the disease as *septicemia anserum exsudativa* [9]. The species epithet is derived from the Latin noun 'anas/atis' meaning 'duck', and the Latin adjective 'pestifer' meaning 'pestilence-carrying' referring to the pathogenic effect the species has on water-fowl, especially ducks. The type strain of the species was isolated from blood of ducklings on Long Island, New York, and identified by Bruner in 1954 [3]. Further isolates were obtained from all kinds of avian hosts, however, pigeons and mammals are not infected by this species. *R. anatipestifer* is distributed worldwide and causes serious problems in agricultural flocks of duck, goose and turkey [10], but it has also been found in the upper respiratory tract of clinically healthy birds [7]. There is no indication that the organism can survive outside of its host. Currently, there are two species in the genus *Riemerella*. The only other validly published species of the genus is *R. columbina* which is mainly associated with respiratory disease in pigeons [11]. Here we present a summary classification and a set of features for *R. anatipestifer* ATCC 11845^T^, together with the description of the complete genomic sequencing and annotation.

## Classification and features

A representative genomic 16S rRNA sequence from strain ATCC 11845^T^ was compared using NCBI BLAST under default settings (e.g., considering only the high-scoring segment pairs (HSPs) from the best 250 hits) with the most recent release of the Greengenes database [12] and the relative frequencies, weighted by BLAST scores, of taxa and keywords (reduced to their stem [13]) were determined. The five most frequent genera were *Riemerella* (79.2%), *Chryseobacterium* (17.1%), *Bergeyella* (2.6%), “*Rosa”* (0.6%; misnomer) and *Cloacibacterium* (0.5%) (166 hits in total). Regarding the 124 hits to sequences from members of the species, the average identity within HSPs was 99.5%, whereas the average coverage by HSPs was 95.2%. Among all other species, the one yielding the highest score was “*Rosa chinensis”*, apparently a severe misannotation, which corresponded to an identity of 99.8% and an HSP coverage of 91.9%. The highest-scoring environmental sequence was EF219033 ('structure and significance *Rhizobiales* biofouling biofilms on reverse osmosis membrane treating MBR effluent clone RO224'), which showed an identity of 96.0% and a HSP coverage of 97.8%. The five most frequent keywords within the labels of environmental samples which yielded hits were 'skin' (10.3%), 'human' (4.9%), 'biota, cutan, lesion, psoriat' (4.0%) and 'fossa' (4.0%) (84 hits in total). Environmental samples which yielded hits of a higher score than the highest scoring species were not found.

**Figure 1:**
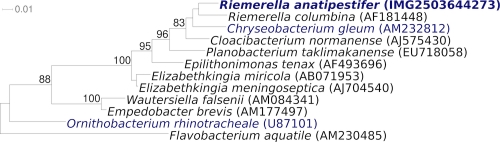
Phylogenetic tree highlighting the position of *R. anatipestifer* relative to a selection of the other type strains within the family *Flavobacteriaceae*. The tree was inferred from 1,391 aligned characters [14,15] of the 16S rRNA gene sequence under the maximum likelihood criterion [16] and rooted with the type strain of the family *Flavobacteriaceae*. The branches are scaled in terms of the expected number of substitutions per site. Numbers above branches are support values from 750 bootstrap replicates [17] if larger than 60%. Lineages with type strain genome sequencing projects registered in GOLD [18] are shown in blue, published genomes in bold.

Figure 1 shows the phylogenetic neighborhood of *R. anatipestifer* in a 16S rRNA based tree. The sequences of the three identical 16S rRNA gene copies in the genome differ by one nucleotide from the previously published 16S rRNA sequence (U60101).

**Figure 2:**
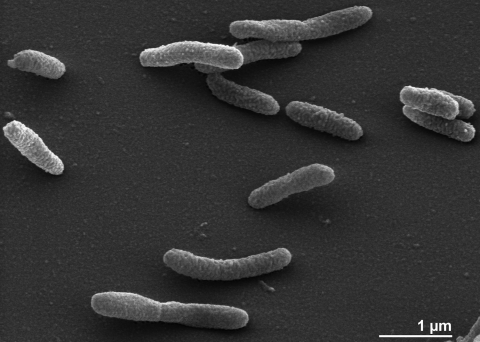
Scanning electron micrograph of *R. anatipestifer* ATCC 11845^T^

The cells of *R. anatipestifer* are generally rod-shaped (0.3-0.5 × 1.0-2.5 µm) with round ends (Figure 2) [6]. *R. anatipestifer* is a Gram-negative, non spore-forming bacterium (Table 1). The organism is described as non-motile. Gliding motility is not observed. Only three genes associated with motility have been found in the genome (see below). The organism is a capnophilic chemoorganotroph which prefers microaerobic conditions for growth. The optimum temperature for growth is 37°C, most strains can grow at 45°C but not at 4°C. Catalase and oxidase are present, thiamine is required for growth [6]. *R. anatipestifer* is not able to reduce nitrate and does not produce hydrogen sulfide. The organism tolerates 10% bile in serum but no growth occurs on agar containing 40% bile in serum [6]. Many biochemical reactions are negative or strain-dependent: Hinz *et al.* have stated that “*R. anatipestifer* is not easy to identify because it is characterized more by the absence than by the presence of specific biochemical properties” [31]. The organism has proteolytic activity but its capacity to utilize carbohydrates is strain-dependent and has been discussed controversially. It has been described that carbohydrates are used oxidatively and that *R. anatipestifer* is able to produce acid from glucose and maltose, less often from fructose, dextrin, mannose, trehalose, inositol, arabinose and rhamnose [31]. The production of indole is strain-dependent; the type strain does not produce indole [6]. Esculin is not hydrolyzed by most *R. anatipestifer* strains, a trait useful for distinguishing these strains from *R. columbina* strains [32]. Strain ATCC 11845^T^ exhibits positive reactions for alkaline and acid phosphatase, ester lipase C8, leucine arylamidase, valine arylamidase, cystine arylamidase, phophoamidase, α-glucosidase and esterase C4. It does not produce α- and β-galactosidases, β-glucuronidase, β-glucosidase, α-mannosidase, β-glucosaminidase, lipase C14, fucosidase, trypsin, ornithine and lysine decarboxylases and phenylalanine deaminase [6].

**Table 1:** Classification and general features of *R. anatipestifer* ATCC 11845^T^ according to the MIGS recommendations [19].

MIGS ID	Property	Term	Evidence code
	Current classification	Domain *Bacteria*	TAS [20]
		Phylum *Bacteroidetes*	TAS [21,22]
		Class '*Flavobacteria*'	TAS [21,23]
		Order '*Flavobacteriales*'	TAS [21,24]
		Family *Flavobacteriaceae*	TAS [21,25-28]
		Genus *Riemerella*	TAS [6,11]
		Species *Riemerella anatipestifer*	TAS [6]
		Type strain ATCC 11845	NAS
	Gram stain	negative	TAS [3]
	Cell shape	rod-shaped with rounded ends, single or in pairs	TAS [3]
	Motility	non-motile	TAS [3]
	Sporulation	none	TAS [6]
	Temperature range	mesophile	TAS [6]
	Optimum temperature	37°C	TAS [3]
	Salinity	normal	NAS
MIGS-22	Oxygen requirement	microaerobic	TAS [3]
	Carbon source	proteins	TAS [6]
	Energy source	chemoorganotroph	TAS [6]
MIGS-6	Habitat	waterfowl and other birds	TAS [6]
MIGS-15	Biotic relationship	symbiotic	TAS [6]
MIGS-14	Pathogenicity	septicemia	TAS [6]
	Biosafety level	2	TAS [29]
	Isolation	duck blood	TAS [3]
MIGS-4	Geographic location	Long Island, New York, USA	TAS [3]
MIGS-5	Sample collection time	1954	TAS [3]
MIGS-4.1	Latitude	not reported	
MIGS-4.2	Longitude	not reported	
MIGS-4.3	Depth	not reported	
MIGS-4.4	Altitude	not reported	

Evidence codes - IDA: Inferred from Direct Assay (first time in publication); TAS: Traceable Author Statement (i.e., a direct report exists in the literature); NAS: Non-traceable Author Statement (i.e., not directly observed for the living, isolated sample, but based on a generally accepted property for the species, or anecdotal evidence). These evidence codes are from of the Gene Ontology project [30]. If the evidence code is IDA, then the property was directly observed by one of the authors or an expert mentioned in the acknowledgements.

*R. anatipestifer* is generally susceptible to enrofloxacin, amoxicillin, chloramphenicol, novobiocin, spiramycin, lincomycin and tetracyclines. Antibiotic resistance of the organism is steadily increasing: resistance to penicillin G, streptomycin and sulfonamides has been reported and more than 90% of all strains are resistant to polymyxin B, colistin, gentamycin, neomycin and kanamycin [33]. The transmission of *R. anatipestifer* in ducks occurs vertically through the egg as well as horizontally via the respiratory route. The disease affects primarily young ducks where it typically involves the respiratory tract and nervous system. Ocular and nasal discharge are often typical for the onset of the disease, lameness can be observed at a later state. The mortality ranges between 1 and 10%, surviving animals may be stunted [34,35]. Vaccination of flocks has proven a valuable course of protection, however, immunity is serovar-specific and more than 20 serovars of *R. anatipestifer* are known [36]. It is remarkable that *R. anatipestifer* persists post-infection on duck farms; biofilm formation of the organism is discussed as one possible explanation [37].

### Chemotaxonomy

Few data are available for *R. anatipestifer* strain ATCC 11845^T^. The sole respiratory quinone found in this species is menaquinone [38]. Which specific quinone is present in *R. anatipestifer* remains unclear from the literature: whereas Segers *et al*. specify menaquinone 7 as sole respiratory quinone in the type strain [6], Vancanneyt *et al*. claim menaquinone 6 is the major respiratory quinone of the type species [11]. Typically, representatives of the genus *Riemerella* contain branched-chain fatty acids in high percentages. Major fatty acids of *R. anatipestifer* are *iso-*C_15:0_ (50-60%), *iso-*C_13:0_ (15-20%), 3-hydroxy *iso-*C_17:0_ (13-18%), 3-hydroxy *anteiso-*C_15:0_ (8-11%) and *anteiso-*C_15:0_ (6-8%) [6].

## Genome sequencing and annotation

### Genome project history

This organism was selected for sequencing on the basis of its phylogenetic position [39], and is part of the *** G****enomic* *** E****ncyclopedia of* *** B****acteria and* *** A****rchaea * project [40]. The genome project is deposited in the Genomes On Line Database [18] and the complete genome sequence is deposited in GenBank. Sequencing, finishing and annotation were performed by the DOE Joint Genome Institute (JGI). A summary of the project information is shown in Table 2.

**Table 2:** Genome sequencing project information

**MIGS ID**	**Property**	**Term**
MIGS-31	Finishing quality	Finished
MIGS-28	Libraries used	Three genomic libraries: one 454 pyrosequence standard library, one 454 PE library (14 kb insert size), one Illumina library
MIGS-29	Sequencing platforms	Illumina GAii, 454 GS FLX Titanium
MIGS-31.2	Sequencing coverage	431 × Illumina; 77.6 × pyrosequence
MIGS-30	Assemblers	Newbler version 2.3, Velvet 0.7.63, phrap SPS - 4.24
MIGS-32	Gene calling method	Prodigal 1.4, GenePRIMP
	INSDC ID	CP002346
	Genbank Date of Release	December 2, 2010
	GOLD ID	Gc01548
	NCBI project ID	41989
	Database: IMG-GEBA	2503538031
MIGS-13	Source material identifier	DSM 15868
	Project relevance	Tree of Life, GEBA

### Growth conditions and DNA isolation

*R. anatipestifer* ATCC 11845^T^, DSM 15868, was grown microaerobically in DSMZ medium 535 (Trypticase Soy Broth Medium) [41] at 37°C. DNA was isolated from 0.5-1 g of cell paste using MasterPure Gram-positive DNA purification kit (Epicentre MGP04100) following the standard protocol as recommended by the manufacturer, with modification st/DL for cell lysis as described in Wu *et al*. [40]. DNA is available through the DNA Bank Network [42].

### Genome sequencing and assembly

The draft genome was generated at the DOE Joint Genome Institute (JGI) using a combination of Illumina and 454 technologies (Roche). For this genome, we constructed and sequenced an Illumina GAii shotgun library which generated 26,937,600 reads totaling 969.8 Mb, a 454 Titanium standard library which generated 238,617 reads and a paired end 454 library with an average insert size of 14.3 kb which generated 112,671 reads totaling 141.8 Mb of 454 data. All general aspects of library construction and sequencing performed at the JGI can be found at [43]. The initial draft assembly contained 28 contigs in one scaffold. The 454 Titanium standard data and the 454 paired end data were assembled together with Newbler, version 2.3. The Newbler consensus sequences were computationally shredded into 2 kb overlapping fake reads (shreds). Illumina sequencing data was assembled with VELVET, version 0.7.63 [44], and the consensus sequences were computationally shredded into 1.5 kb overlapping fake reads (shreds). We integrated the 454 Newbler consensus shreds, the Illumina VELVET consensus shreds and the read pairs in the 454 paired end library using parallel phrap, version SPS - 4.24 (High Performance Software, LLC). The software Consed [45] was used in the following finishing process. Illumina data was used to correct potential base errors and increase consensus quality using the software Polisher developed at JGI [46]. Possible mis-assemblies were corrected using gapResolution [43], Dupfinisher [47], or sequencing cloned bridging PCR fragments with subcloning. Gaps between contigs were closed by editing in Consed, by PCR and by Bubble PCR (J-F Cheng, unpublished) primer walks. A total of 388 additional reactions were necessary to close gaps and to raise the quality of the finished sequence. The error rate of the completed genome sequence is less than 1 in 100,000. Together, the combination of the Illumina and 454 sequencing platforms provided 508.6 × coverage of the genome.

### Genome annotation

Genes were identified using Prodigal [48] as part of the Oak Ridge National Laboratory genome annotation pipeline, followed by a round of manual curation using the JGI GenePRIMP pipeline [49]. The predicted CDSs were translated and used to search the National Center for Biotechnology Information (NCBI) nonredundant database, UniProt, TIGR-Fam, Pfam, PRIAM, KEGG, COG, and InterPro databases. Additional gene prediction analysis and functional annotation was performed within the Integrated Microbial Genomes - Expert Review (IMG-ER) platform [50].

## Genome properties

**Figure 3:**
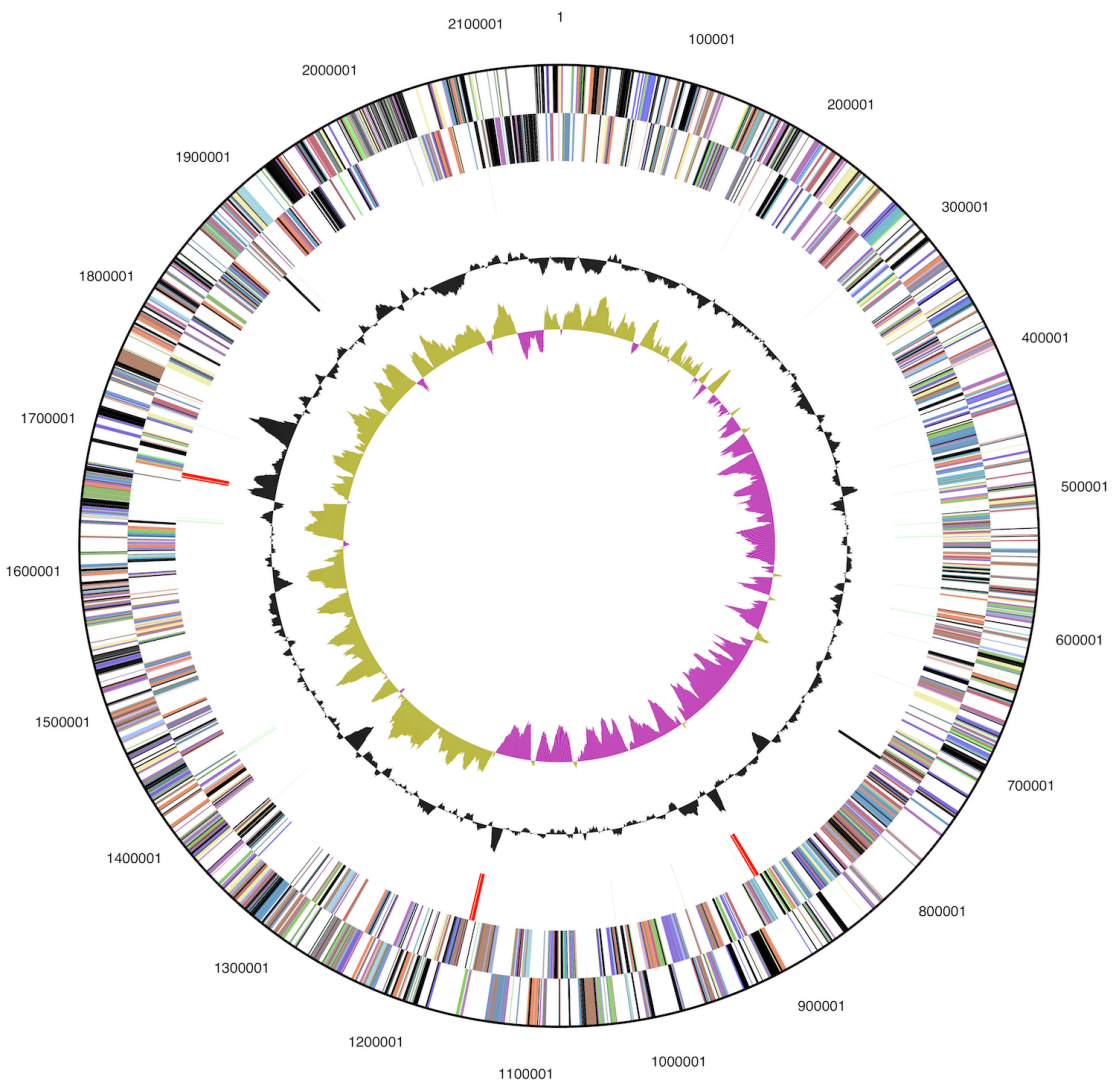
Graphical circular map of the chromosome. From outside to the center: Genes on forward strand (color by COG categories), Genes on reverse strand (color by COG categories), RNA genes (tRNAs green, rRNAs red, other RNAs black), GC content, GC skew.

The genome consists of a 2,155,121 bp long chromosome with a G+C content of 35.0% (Table 3 and Figure 3). Of the 2,052 genes predicted, 2,001 were protein-coding genes, and 51 RNAs; 29 pseudogenes were also identified. The majority of the protein-coding genes (64.1%) were assigned with a putative function while the remaining ones were annotated as hypothetical proteins. The distribution of genes into COGs functional categories is presented in Table 4.

**Table 3:** Genome Statistics

**Attribute**	**Value**	**% of Total**
Genome size (bp)	2,155,121	100.00%
DNA coding region (bp)	1,948,611	90.42%
DNA G+C content (bp)	754,510	35.01%
Number of replicons	1	
Extrachromosomal elements	0	
Total genes	2,052	100.00%
RNA genes	51	2.49%
rRNA operons	3	
Protein-coding genes	2,001	97.51%
Pseudo genes	29	1.41%
Genes with function prediction	1,316	64.10%
Genes in paralog clusters	125	6.09%
Genes assigned to COGs	1,283	64.13%
Genes assigned Pfam domains	1,411	68.76%
Genes with signal peptides	472	23.00%
Genes with transmembrane helices	414	20.18%
CRISPR repeats	2	

**Table 4:** Number of genes associated with the general COG functional categories

**Code**	**value**	**%age**	**Description**
J	133	9.7	Translation, ribosomal structure and biogenesis
A	0	0.0	RNA processing and modification
K	70	5.1	Transcription
L	92	6.7	Replication, recombination and repair
B	0	0.0	Chromatin structure and dynamics
D	19	1.4	Cell cycle control, cell division, chromosome partitioning
Y	0	0.0	Nuclear structure
V	22	1.6	Defense mechanisms
T	32	2.3	Signal transduction mechanisms
M	139	10.2	Cell wall/membrane/envelope biogenesis
N	3	0.2	Cell motility
Z	0	0.0	Cytoskeleton
W	0	0.0	Extracellular structures
U	26	1.9	Intracellular trafficking, secretion, and vesicular transport
O	66	4.8	Posttranslational modification, protein turnover, chaperones
C	77	5.6	Energy production and conversion
G	39	2.9	Carbohydrate transport and metabolism
E	101	7.4	Amino acid transport and metabolism
F	52	3.8	Nucleotide transport and metabolism
H	83	6.1	Coenzyme transport and metabolism
I	56	4.1	Lipid transport and metabolism
P	88	6.4	Inorganic ion transport and metabolism
Q	20	1.5	Secondary metabolites biosynthesis, transport and catabolism
R	159	11.6	General function prediction only
S	89	6.5	Function unknown
-	769	37.5	Not in COGs
